# Extraluminal Colonic Adenocarcinoma Directly Invading the Left Kidney: A Rare Case of Direct Invasion

**DOI:** 10.7759/cureus.114351

**Published:** 2026-08-11

**Authors:** Apostolos Krikelis, Konstantinos Stratakis, Lampros Fountoulis, Nikolaos Korkanas, Andreas Zevlas, Dimitrios Bouklas

**Affiliations:** 1 3rd Department of General Surgery, General Hospital of Athens "G. Gennimatas", Athens, GRC; 2 General Surgery, Sion Cantonal Hospital, Sion, CHE; 3 Department of Pathology, General Hospital of Athens "G. Gennimatas", Athens, GRC

**Keywords:** bowel obstruction, case report, colon cancer, descending colon, gerota fascia, left kidney, multivisceral resection, nephrectomy, renal invasion, subtotal colectomy

## Abstract

The most common distant metastatic sites of colorectal cancer (CRC) are the liver, lungs, and peritoneum. Renal involvement from colon cancer is rare and, when present, usually occurs via hematogenous spread. Direct invasion of the kidney by a primary colonic adenocarcinoma is exceptionally uncommon, with very few cases reported in the literature. We report the case of a 65-year-old woman who presented with vomiting, obstipation, and diffuse abdominal pain due to bowel obstruction. Computed tomography (CT) of the abdomen demonstrated soft tissue thickening around the mid-descending colon, partial luminal obstruction, proximal bowel dilatation with air-fluid levels, marked cecal dilatation, and suspected direct contact with the left kidney. Emergency rectosigmoidoscopy revealed an obstructing mass in the descending colon. The patient subsequently underwent urgent exploratory laparotomy, during which marked colonic dilatation with impending cecal rupture was identified. Subtotal colectomy with end ileostomy was performed. Intraoperatively, the tumor was also found to infiltrate the left kidney, and left nephrectomy was carried out. Histopathological examination established the diagnosis of moderately differentiated colonic adenocarcinoma measuring 6.6 cm, with direct invasion into Gerota’s fascia, the perirenal adipose tissue, and the renal parenchyma. This case highlights an exceptionally rare pattern of locally advanced colon cancer and underscores the need to consider adjacent organ invasion in patients presenting with obstructing left-sided colonic tumors.

## Introduction

Colorectal cancer (CRC) is one of the leading causes of cancer-related morbidity and mortality worldwide [1]. According to the Global Cancer Statistics 2020, more than 1.9 million new CRC cases and 0.9 million deaths were estimated globally [2]. The most frequent metastatic sites of colon cancer are the liver, lungs, and peritoneum, whereas renal involvement is extremely rare. A postmortem study of more than 10,000 patients who died from malignant disease demonstrated that less than 3% of secondary renal neoplasms originated from primary colon cancer [3]. In the few reported cases, renal involvement has usually occurred via hematogenous spread [4-6]. To date, there is only one case in the literature reporting the direct invasion of a colonic adenocarcinoma into the kidney [7]. We present a rare case of extraluminal adenocarcinoma of the descending colon directly invading the left kidney.

## Case presentation

**Figure 1:**
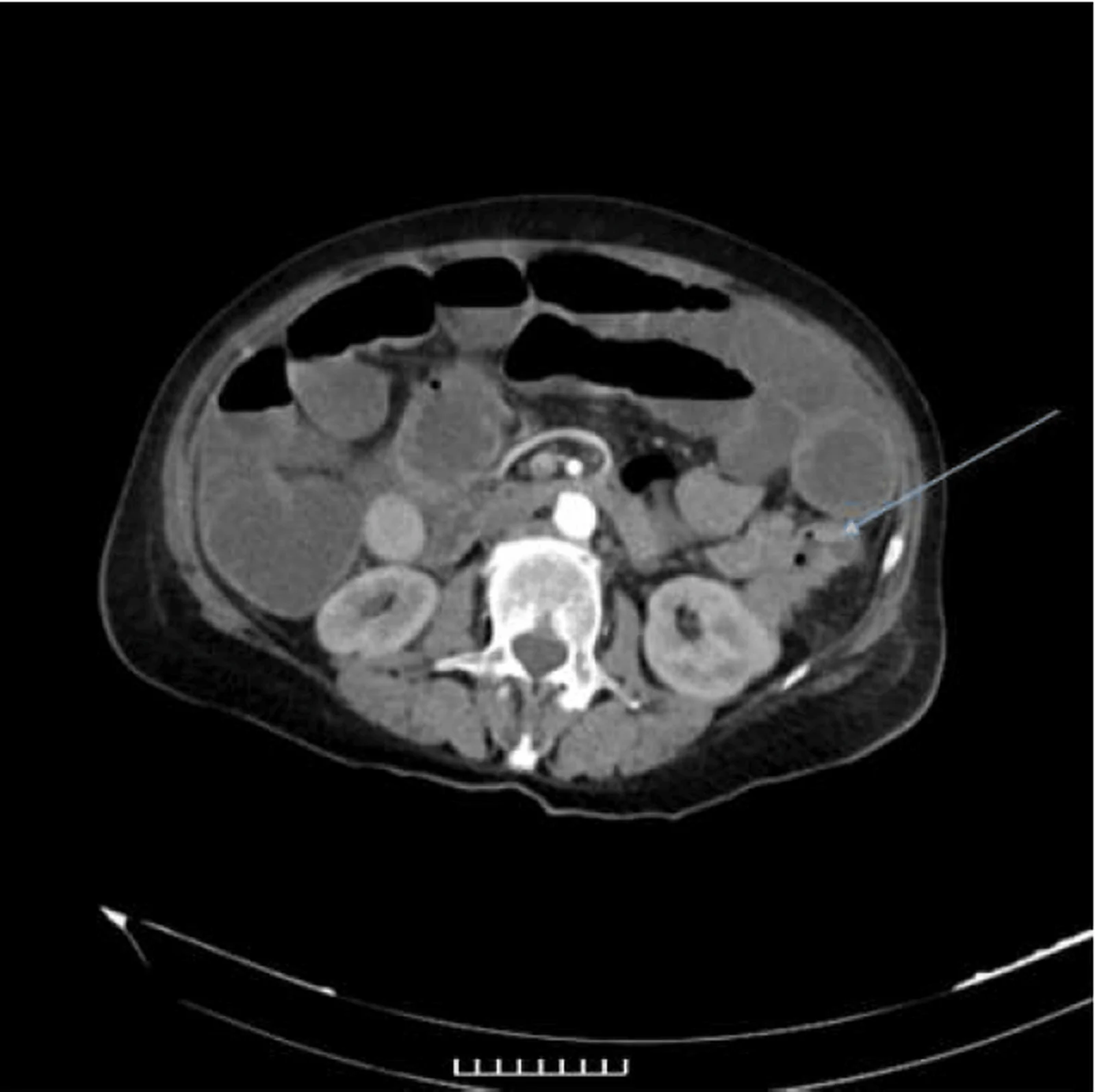
Axial abdominal CT image demonstrating a descending colon mass with retroperitoneal extension and suspected direct contact with the left kidney (arrow)

A 65-year-old woman was admitted to the Surgical Emergency Department of the General Hospital of Athens “G. Gennimatas” on 6 October 2022 with vomiting, obstipation, and abdominal pain. Computed tomography (CT) of the upper and lower abdomen revealed soft tissue surrounding the mid-portion of the descending colon, causing incomplete luminal obstruction, with bowel distension and air-fluid levels involving the more proximal colon and loops of small intestine up to the level just proximal to the horizontal portion of the duodenum. Marked cecal dilatation was also noted, measuring 9 cm (Figure 1). In addition, the lesion appeared to be in direct contact with the left kidney. Perihepatic and perisplenic fluid collections were also described.

The patient underwent emergency rectosigmoidoscopy, which demonstrated a mass in the descending colon causing luminal obstruction. Following admission to the 3rd Surgical Department, she was taken urgently to the operating room for exploratory laparotomy. Intraoperatively, a descending colon mass with posterior peritoneal fixation was identified. The proximal colon was markedly dilated, with a threatened cecum and impending rupture. Furthermore, the tumor was found to infiltrate the left kidney and adjacent tissues. In view of these findings, subtotal colectomy with end ileostomy was performed, and left nephrectomy was carried out by the on-call urologist.

The postoperative course was generally uneventful. Oral intake was tolerated well. Cytological examination of the peritoneal fluid was negative. The patient was discharged in good clinical condition on 14 October 2022.

Pathology evaluation

**Figure 2:**
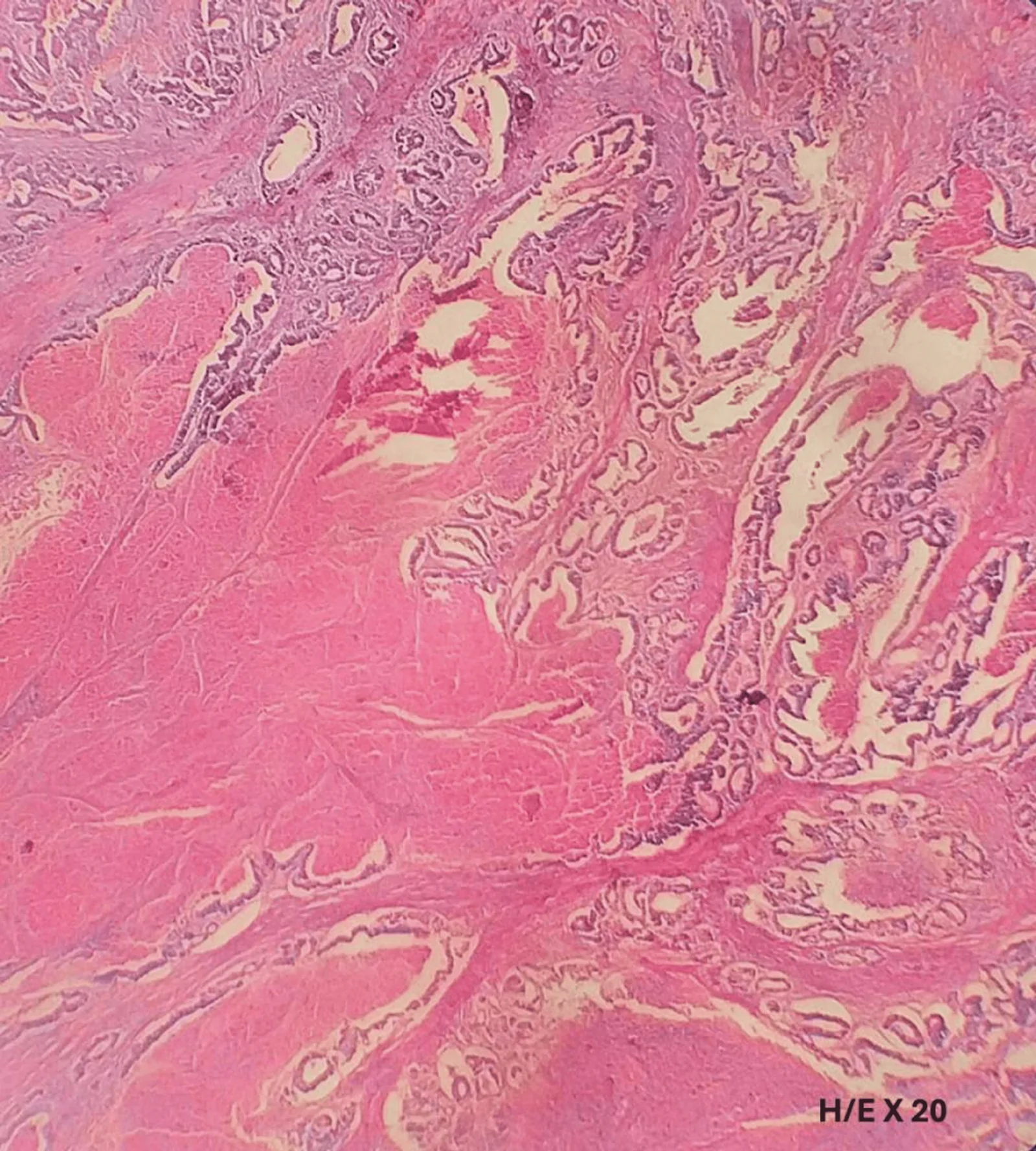
Histopathological image showing colonic adenocarcinoma with transmural invasion

**Figure 3:**
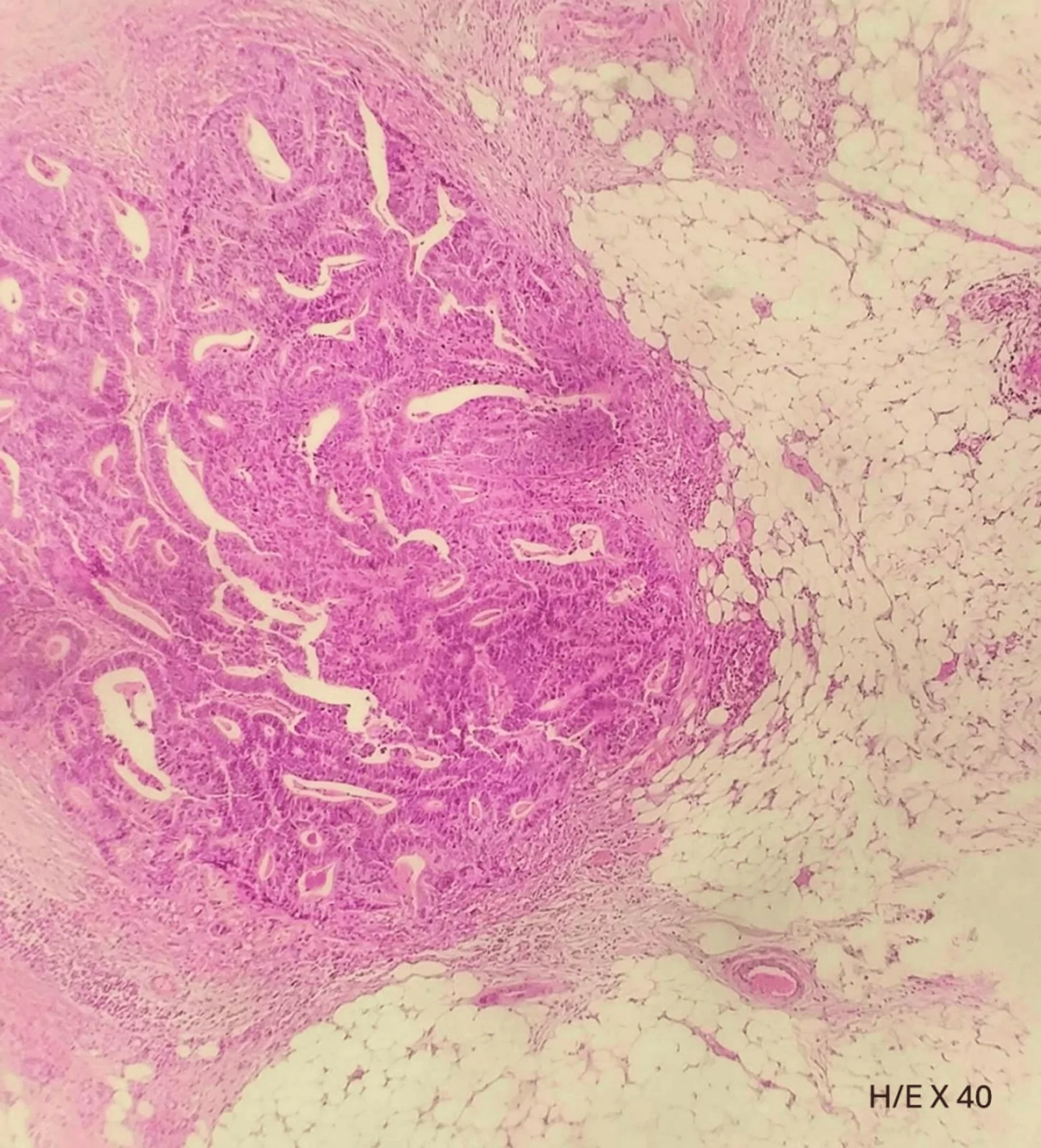
Histopathological image showing perirenal fat invasion

**Figure 4:**
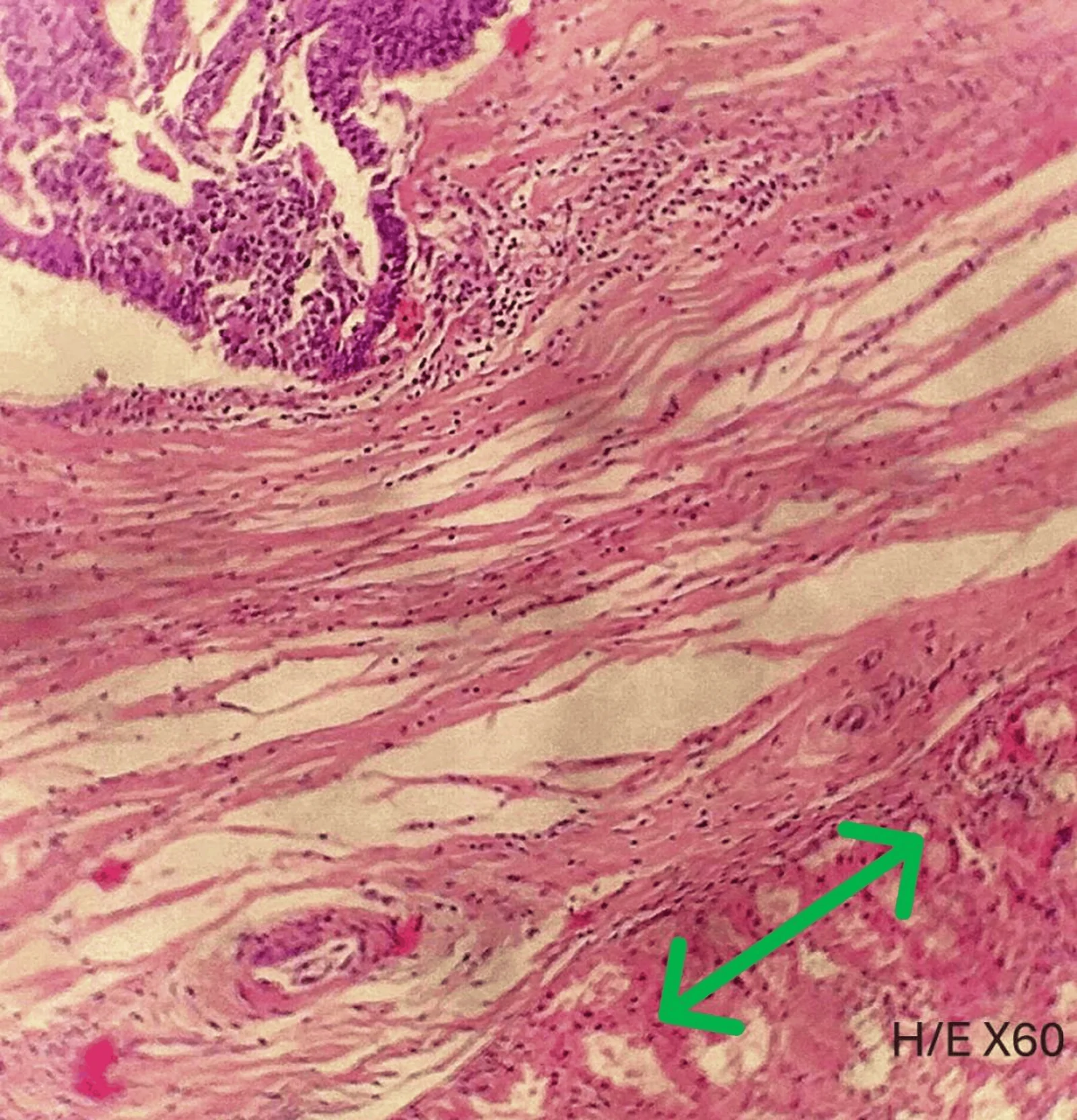
Histopathological image showing intrarenal invasion

Histopathological examination established the diagnosis of moderately differentiated colonic adenocarcinoma with a maximum diameter of 6.6 cm and transmural invasion (Figure 2). Examination of the nephrectomy specimen demonstrated direct invasion of the primary colonic tumor into the anterior renal fascia (Gerota’s fascia), the perirenal adipose tissue (Figure 3), and the renal parenchyma (Figure 4). These findings confirmed direct contiguous extension of the colonic adenocarcinoma into the left kidney rather than distant renal metastasis. 

## Discussion

Renal involvement in colon cancer is rare and, when present, is usually attributed to hematogenous dissemination. It was initially described in a study including autopsies [3]. Direct invasion of the kidney by a primary colonic carcinoma is exceptionally uncommon. To the best of our knowledge, of the studies published in the literature, only one reports the case of an extraluminal colonic adenocarcinoma directly invading the kidney [7]. The rarity of this pattern of spread may be explained by the presence of anatomical barriers between the colon and the kidney, particularly the peritoneum and Gerota’s fascia, which normally serve a critical role in preventing the spread of disease [8]. Today, due to significant improvements in imaging techniques (such as CT scans) and health screening tests, diagnosis of secondary kidney tumors is more frequent. However, the discovery of these tumors is mostly incidental and requires histopathological examination, since they are usually asymptomatic and of small size. In general, kidneys are highly vascularized organs, and in the majority of cases metastasis is a result of the primary tumor spreading through the hematogenous pathway [9].

Our case is notable for two reasons. First, it represents a highly unusual pattern of locally advanced descending colon adenocarcinoma with direct renal invasion. Second, it presented as a surgical emergency with bowel obstruction, severe proximal colonic dilatation, and impending cecal rupture, ultimately requiring urgent multivisceral resection. These features increase the clinical importance of the case, as recognition of possible adjacent organ invasion in left-sided obstructing colon tumors may affect both operative planning and intraoperative decision-making.

In this setting, en bloc resection remains the cornerstone of treatment whenever technically feasible and oncologically appropriate. This approach is consistent with current surgical guidelines, which recommend complete en bloc resection with negative margins for resectable colon cancers that adhere to or invade adjacent organs when curative treatment is intended [10]. However, nephrectomy adds considerable operative complexity and may increase perioperative morbidity. For that reason, preoperative suspicion of renal invasion is particularly valuable. In the present patient, CT had already suggested direct contact between the descending colon lesion and the left kidney, a finding later confirmed intraoperatively and histologically.

Our case therefore adds to the limited published experience regarding direct renal invasion by primary colon cancer and supports the view that this rare mode of spread should be considered in selected patients with bulky left-sided colonic tumors and retroperitoneal extension.

## Conclusions

Direct invasion of the kidney by colonic adenocarcinoma is an exceptionally rare manifestation of locally advanced colon cancer. This case highlights the importance of considering adjacent organ invasion in patients presenting with obstructing left-sided colonic tumors and radiologic evidence of retroperitoneal extension or renal contact. Early recognition of this possibility may facilitate appropriate surgical planning, and urgent multivisceral resection may be required in the emergency setting to achieve adequate disease control.
